# Evaluation and optimization of the Circulating Cathodic Antigen (POC-CCA) cassette test for detecting *Schistosoma mansoni* infection by using image analysis in school children in Mwanza Region, Tanzania

**DOI:** 10.1016/j.parepi.2016.04.002

**Published:** 2016-04-22

**Authors:** Miriam Casacuberta, Safari Kinunghi, Birgitte J. Vennervald, Annette Olsen

**Affiliations:** a Parasitology and Aquatic Diseases, Department of Veterinary Disease Biology, University of Copenhagen, Dyrlaegevej 100, DK-1870 Frederiksberg C, Denmark; b National Institute for Medical Research (NIMR), Mwanza Research Centre, Mwanza, Tanzania)

**Keywords:** *Schistosoma mansoni*, School children, CCA, Computer image analyses, Tanzania

## Abstract

There is a need for diagnostic techniques which are sensitive, specific, rapid and easy to perform at the point-of-care. The aim of this study was to evaluate the diagnostic performance of the Circulating Cathodic Antigen (POC-CCA) assay for *Schistosoma mansoni* in four schools along the coast of Lake Victoria in Mwanza Region, Tanzania, and to optimize the reading of the POC-CCA test lines by using a computer software image analysis. Initially, a pilot study in 106 school children indicated that time of urine collection did not have an impact on CCA results as 84.9% (90) had identical scores from a urine collected in the morning and a urine taken at midday after drinking 0.5 L of water. The main study was conducted among 404 school children (aged 9–12 years) where stool and urine samples were collected for three consecutive days. For *S. mansoni* diagnosis, stool samples were examined for eggs with duplicate Kato-Katz smears, whereas urine samples were tested for presence of antigen by POC-CCA. The proportion of positive individuals for *S. mansoni* by one POC-CCA was higher compared to two Kato-Katz smears (66.1% vs. 28.7%; p < 0.0001). Both proportions increased expectedly when three POC-CCAs were compared to six Kato-Katz smears (75.0% vs. 42.6%; p < 0.0001). Three POC-CCAs were more sensitive (94.7%) than six Kato-Katz smears (53.8%) using the combined results of three POC-CCAs and six Kato-Katz smears as the ‘gold standard’. To optimize the reading of the POC-CCA, a Software tool (Image Studio Lite®) was used to read and quantify the colour (expressed as pixels) of the test line on all positive tests, showing a positive correlation between number of pixels and the visually scored intensities and between number of pixels and egg counts. In conclusion, the POC-CCA assay seems to be a more appropriate tool for *S. mansoni* diagnosis compared to the Kato-Katz method in endemic communities such as Mwanza Region. Optimization of the tool in terms of cassette-reading could be assessed by computer software which was able to quantify the colour of the lines in the strip of the cassette.

## 1 Introduction

Progress has been done in control of schistosomiasis over the last 20 years (Chitsulo et al., 2000) where prevalence of infection has been reduced to lower levels. However, it appears difficult to estimate the accurate prevalence of infection when there is a lack of sensitive diagnostic tools (Erko et al., 2013, Shane et al., 2011) especially when light intensity is present in a study area (Berhe et al., 2004).

Currently, the World Health Organization (WHO) recommends detection of eggs in stool by the Kato-Katz technique (Katz et al., 1972) as the standard tool for the qualitative and quantitative diagnosis of *Schistosoma mansoni* infection. It is the most direct and specific technique by which the presence of a schistosome infection can be established (Hamilton et al., 1998) and has been chosen due to its assumed high specificity of 100% (Doenhoff et al., 2004, Gray et al., 2011), relative simple performance under field conditions and in places with limited health facilities, such as in Sub-Saharan Africa, as well as low cost compared to currently available diagnostic tests (Kongs et al., 2001, WHO, 2002).

However, it has been widely recognized that the Kato-Katz method lacks sensitivity (Ruiz-Tiben et al., 1979, Gryseels and de Vlas, 1996) especially in situations where light intensity is common (Berhe et al., 2004). These light infections are then missed and become a potential source of *S. mansoni* transmission (Erko et al., 2013). Although the sensitivity of the technique can be improved by increasing the number of stool samples tested (Booth et al., 2003), getting stool samples from individuals on several days is challenging even when sampling takes place in schools because opening-hours and children's timetable have to be considered. In addition, the Kato-Katz method exposes technicians handling fresh stool samples to harmful infectious agents other than schistosomes (Shane et al., 2011). Furthermore, well-trained laboratory technicians are needed in order to perform the test correctly.

Several authors have described and discussed alternative methods to the Kato-Katz technique (Engels, 1997, King, 2001, Barda et al., 2013) among which are the immunological techniques which detect parasite antigens in the host's bodily fluids. Since the early 90-ies, detection of Circulating Cathodic Antigen (CCA) in urine of patients has been widely investigated for diagnosis of *S. mansoni* (Deelder et al., 1980, De Jonge et al., 1990, van Lieshout et al., 2000, van Dam et al., 2004), and during the last 10 years a rapid lateral flow strip has become commercially available as a point-of-care Circulating Cathodic Antigen (POC-CCA) test.

CCA is a genus-specific glycoprotein excreted by young and adult schistosome worms into the circulation of its definitive host. It is positively charged at neutral pH, hence its name cathodic, according to its electrophoretic mobility (Deelder et al., 1980). The POC-CCA test is based on direct detection of parasite CCA in urine using labelled monoclonal antibodies (van Dam et al., 2004) and it provides results of the presence of a worm infection within a few minutes (Standley et al., 2010).

The POC-CCA has the potential to provide more sensitive and rapid detection of intestinal schistosomiasis with already promising results (Shane et al., 2011, Coulibaly et al., 2011) and it has therefore been suggested as an alternative to the Kato-Katz technique (Shane et al., 2011, van Dam et al., 2004, Coulibaly et al., 2011, Stothard et al., 2009). Since CCA is only released from living worms, it is generally assumed that detection of CCA can be interpreted as a reflection of current worm burdens in man (van Dam et al., 1996, Cavalcanti et al., 2013, Rollinson et al., 2013). To support this interpretation, circulating antigens disappear from the urine of schistosomiasis patients within a couple of weeks after successful treatment (Coulibaly et al., 2013, Lamberton et al., 2014).

CCA cassettes are portable and easy to use in the field and do not require a lot of staff training for a proper performance (Stothard et al., 2006, Adriko et al., 2014). In addition, urine collection is easier and less invasive than collection of stool or blood (Doenhoff et al., 2004). The test has been found to be highly sensitive and specific in detecting CCA of *S. mansoni* in high endemic areas (van Dam et al., 2004, Stothard et al., 2006). The sensitivity of the CCA assay ranges from 65 to 100%, largely depending on intensity of infection of the study population (Colley et al., 2013).

However, some investigators have reported limitations referring to low sensitivity in cases of light intensity of infection (Stothard et al., 2006, Legesse and Erko, 2007) and inter-reader variability when it comes to band interpretation of the cassette (Standley et al., 2010). For example, inter-reader variability is especially an issue regarding the ‘trace’ score readings. Because of this it would be useful to develop a tool which can quantify the cassette scores in an objective way.

The overall objective of the present study was to evaluate the diagnostic performance of the POC-CCA urine-cassette test for detecting *S. mansoni* infection in school children from rural districts in Mwanza Region, Tanzania, and to optimize the method further by using a computer software tool for reading the intensity of the test lines on the strips.

## 2 Materials and methods

### 2.1 Ethics statement

The study was part of the ongoing Schistosomiasis Consortium for Operational Research and Evaluation (SCORE) project (years 2011 to 2016), which has been reviewed and approved by the Medical Research Co-ordination Committee (MRCC) of the National Institute for Medical Research (NIMR), Tanzania (ethics clearance certificate no. NIMR/HQ/R.8a/Vol.IX/1022) and the University of Georgia Institutional Review Board, USA (2011-10353-1).

The objectives of the study were explained to families and children and a written consent sheet was provided by the SCORE team. Individual informed consent was obtained from parents/caretakers on behalf of their children. The assent of included children was obtained and a child's refusal to participate was respected. Every child who participated in the study was assigned with an identification number and results were entered and treated confidentially.

Everyone investigated for *S. mansoni*, whether positive or not, was treated with a single dose of praziquantel (40 mg/kg body weight) when SCORE finished field work in August–September 2014. The possible side effects of the medication were explained to children and parents.

### 2.2 Study area and population

The study took place in three rural districts of Mwanza Region in Tanzania, specifically in Misungwi, Sengerema and Magu districts, and was conducted between March and May 2014. The region was chosen because the SCORE project is implemented there. Four different public primary schools were followed (coordinates for the schools in decimal degrees: Kigongo − 2.70977, 32.89865; Chole − 2.72950, 32.94414; Nyamatongo − 2.52114, 32.79054; Nyashimo − 2.37745, 33.58347). The samples were collected from children aged between 9 and 12 years who were selected from the four schools by the SCORE project.

### 2.3 Sample collection

#### 2.3.1 Pilot study

For the pilot study, only urine specimens were collected and tested with POC-CCA cassettes. Two urine samples, one assumed to be the first in the morning and labelled as *morning* and the other sample taken after participants have taken 0.5 L of water, labelled as *midday*, were examined. During collection day, urine containers were kept in a portable fridge at 5 °C for few hours, until the team reached proper facilities. Then, urine samples were stored at − 20 °C until the field work was done and it was possible to test them. All containers were labelled and identified with an ID number.

#### 2.3.2 Main study

For the main study, individuals were asked to provide one stool and one urine samples on three consecutive days. All samples (stool and urine) were collected between 9.00 AM and 13.00 PM. One container for urine and one for stool were provided to the children who were asked to deliver it back to the team within the same morning. During collection day, urines were treated as described above in Section 2.3.1.

### 2.4 Parasitological measurements

#### 2.4.1 Main study

Only in the main study, stool samples were examined for *S. mansoni* eggs with duplicate Kato-Katz thick smears using a 41.7 mg template (Katz et al., 1972). The smears were prepared in the field following the Kato-Katz protocol and kept in microscope slide boxes until they could be examined. The microscope slides were quantitatively examined for intestinal schistosomiasis by four experienced laboratory technicians once the team returned to the laboratory between 1 and 4 days after the field work. A fifth technician checked a random selection of 10% of the readings for discrepancy. In case of discrepancy, slides were recounted until consensus was obtained. Mean number of eggs of the six slides were calculated and multiplied by 24 and expressed as eggs per gram of faeces (EPG).

### 2.5 CCA measurements

#### 2.5.1 Pilot and main study

Urine samples were left to thaw between 2 and 3 h at room temperature, mixed well and investigated for the presence of CCA using a commercially available rapid diagnostic test (POC-CCA, batch no. 33,873, Rapid Medical Diagnostics, Pretoria, South Africa) according to the manufacturer's procedure (http://rapid-diagnostics.com/downloads/RMD%20Pamphlet%202011_06_13%20.pdf). One drop of urine was added to each cassette and after the urine has been absorbed, one drop of buffer was added. The test results were read 20 min after adding of the buffer.

### 2.6 Visual classification of CCA bands

#### 2.6.1 Pilot and main study

A test was considered valid only if the control line turned into dark-pink colour, and in case this didn't happen, the sample was retested using a new cassette. Valid cassettes were classified as either positive or negative, according to the manufacturer's instructions, with further stratification depending on the strength of the colour reaction. Thus, positive cassettes were classified into trace (when the result line was extremely weak), + 1 (when result line was easy to see but still weak), + 2 (when result line was dark, but lighter than the control line) or + 3 (when result line was as dark as or darker than the control line) as done before in other studies (Shane et al., 2011, Coulibaly et al., 2011, Coulibaly et al., 2013, Stothard et al., 2011).

### 2.7 Photography of CCA bands

#### 2.7.1 Main study

In the main study, pictures of all positive cassettes were taken in order to quantify the colour of the bands using a computer tool. A set up was built in order to achieve a standardized method for taking pictures. Thus, a maximum of two POC-CCA cassettes were placed vertically and next to each other, keeping same angle (90°) to the ground and same distance (7 cm) to the camera. In this way, all positive cassettes were photographed under natural light conditions in the laboratory. The camera used was a *Panasonic lumix dmc fx37* and pictures were taken without flash immediately after visual inspection of the colour band.

### 2.8 Quantification of the bands

#### 2.8.1 Main study

**Fig. 1:**
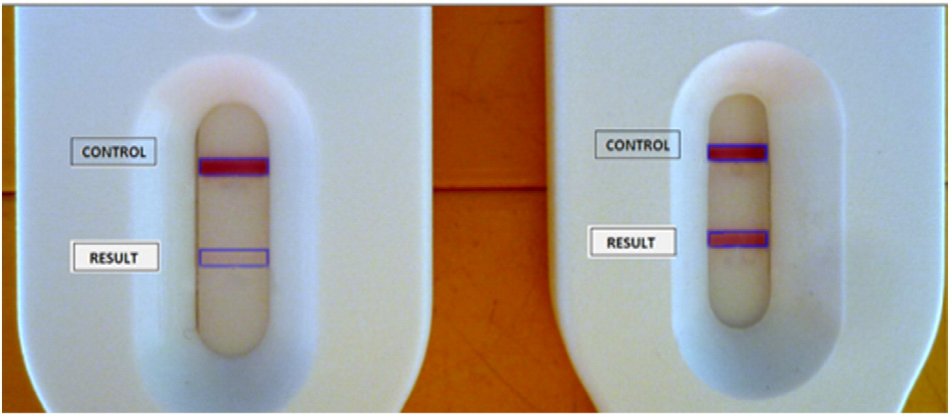
Pixel quantification of two POC-CCA tests. Detailed picture from two cassettes with their control and result bands. To the left, POC-CCA shows a visual ‘trace’ score. To the right, POC-CCA shows a visual + 3.

All pictures were uploaded to the computer in JPEG format. Software-computer tool (*Image Studio Lite Western Blot Analysis Software version 3.1*) was chosen to perform pixel quantification of the images. To represent colour images, separate red, green and blue components (channels) were specified for each pixel, and the pixel value is thus a vector of three numbers. All channels were then selected in order to give the best resolution to the eye (Taylor et al., 2013). Pictures were imported into *Image Studio Lite* and a frame was drawn around the bands that needed to be quantified. *Image Studio Lite* calculates the average of the pixels in the frame for the three channels. To acquire the final value for pixel quantification, a *signal* value was calculated as the sum of the pixel intensities in a frame (in that case, a cassette band) and subtracting the background value. Every *result band* from the cassette was compared with its own *control band* and the proportion between the two bands calculated (Fig. 1). By doing this, the influence of differences in picture quality and amount of light present and the possible variability in colour-intensity between cassettes were negligible. In this way, it became possible to quantify the intensity of the *result band* of the cassettes in relation to the corresponding *control*. It is the calculated proportions (by dividing *result* by *control*) that were compared between different cassette samples.

### 2.9 Statistical analysis

Data were entered into a Microsoft Excel spreadsheet and transferred to IBM SPSS statistics version 22 (IBM, Armonk, NY). Statistical analyses were done in Excel and SPSS.

In the pilot study, the proportion of CCA results that did not change between *morning* and *midday* urine (either positives or negatives on both occasions) was compared with the proportion that did change (from positives to negatives or *vice-versa*) by Chi-square test with Yates's correction. Likewise, other comparisons between proportions in both the pilot and the main study used the same statistical test.

For the main study, only children who had complete data records with six Kato-Katz thick smears (three smears with two slides) and three POC-CCA urine samples were included in the final analysis. As suggested by related literature (Erko et al., 2013, Coulibaly et al., 2011, Coulibaly et al., 2013, Lamberton et al., 2014), a combination of results by three Kato-Katz thick smears (with two slides) and triple POC-CCA tests were set as a ‘gold standard’ in order to assess the sensitivity of the tests. A case was considered positive if one or more of the six Kato-Katz smears or the three POC-CCA cassettes was scored positive. For the POC-CCA, ‘trace’ results were considered as positive according to the manufacturer's instructions. Alternatively, a case was only considered negative if all tests were found negative, thus assuming specificity to be 100% for each test (any *S. mansoni* egg or colour band in the POC-CCA represents infection) (Colley et al., 2013).

The strength of agreement between six Kato-Katz thick smears and three POC-CCA tests with the ‘gold standard’ was assessed by Kappa statistics (ƙ) according to previous studies (Coulibaly et al., 2011, Coulibaly et al., 2013, Tchuem Tchuenté et al., 2012) as follows: k = 0 indicating no agreement; k = 0–0.20 indicating poor agreement; k = 0.21–0.40 indicating fair agreement; k = 0.41–0.60 indicating moderate agreement; k = 0.61–0.80 indicating substantial agreement; and k = 0.81–1.0 indicating almost perfect agreement (Landis and Koch, 1977).

To assess the infection intensity and to obtain a standardized measure, arithmetic mean of *S. mansoni* egg counts per gram of faeces (EPG) from the six Kato-Katz thick smears was calculated (Erko et al., 2013, Coulibaly et al., 2011). For each individual, classification into light (1–99 EPG), moderate (100–399 EPG) and heavy (≥ 400 EPG) infections was calculated based on WHO recommendations (WHO, 2002).

Based on CCA scores, each cassette's result was graded as negative, trace, + 1, + 2 and + 3 by looking at the strength of the band as has been described above (Table 1, column ‘score’). Then, each ‘score’ was given a numerical value (Table 1, column ‘numerical value’). Finally, the sum of the numerical values for the three days was determined (Table 1, column ‘sum up value’). For example, if an individual got trace for day 1 (its value will be 1), negative for day 2 (its value will be 0) and + 1 at day 3 (its value will be 2), then the sum up value for the three days will be 3 (1 + 0 + 2) and this number belongs to a trace result according to Table 1.

**Table 1:** Scoring table showing original visual scores from individual POC-CCA test (1st column), the translation to numerical values (2nd column) and the sum up value for the 3 days (3rd column).

Score	Numerical value	Sum up value (3 days)
Negative	0	0
Trace	1	1–3
+ 1	2	4–6
+ 2	3	7–9
+ 3	4	10–12

For quantification of CCA bands, the average of the ‘signal’ value for each band as well as the proportion for each cassette (*result* value divided by *control* value) was calculated by Excel. Each proportion is referred as ‘pixel density’ for a cassette and their average was calculated for three days. The pixel value obtained from the software is a value between 0 and 1 by default.

Correlations between pixel density (calculated as median for the three days) and POC-CCA categories (calculated as mean for the three days according to colour intensity) were calculated by the non-parametric Spearman rank correlation test. Correlations between pixel density and egg counts (calculated as mean of EPG for three days) were also calculated by Spearman rank correlation test. Differences of p < 0.05 were considered as statistically significant in all analyses.

## 3 Results

### 3.1 Pilot study

*Morning* and *midday* urines were collected from a total of 106 children (aged 9–12) and investigated by POC-CCA. The tests scored positive in 81 *morning* (76.4%), and in 91 *midday* (85.8%) urine samples (χ^2^ (1) = 2.5, p > 0.10). Ninety children (84.9%) had identical scores in their *morning* and *midday* urines (positive or negative on both occasions).

### 3.2 Main study

#### 3.2.1 Proportion of *S. mansoni* infection and intensity

**Fig. 2:**
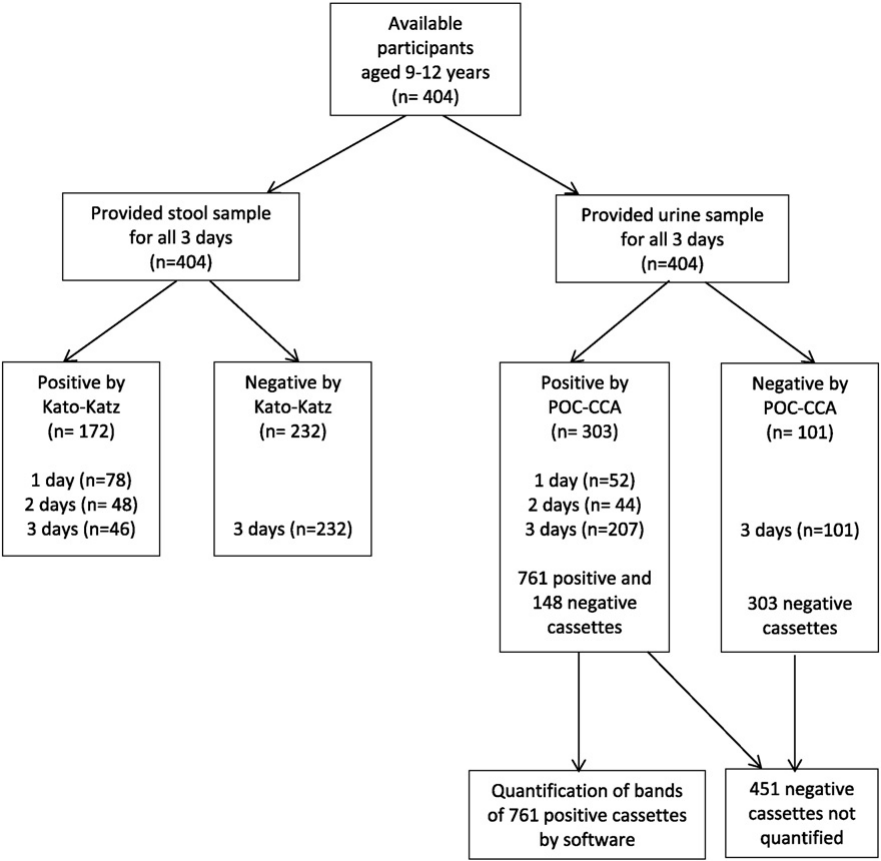
Flow chart of study participation in main study.

Three stool samples and three urine samples were available from 404 children (aged 9–12). A flow chart showing study participation for the main study is presented in Fig. 2. The proportion of individuals infected with *S. mansoni* based on the ‘gold standard’ was 79.2% (Table 2). The proportion was 42.6% if based on six Kato-Katz smears and 75.0% if based on three POC-CCA tests on urines considering trace as a positive result (χ^2^ (1) = 86.3, p < 0.0001). If the two diagnostic methods were compared for the first stool sample only, the proportion of positive individuals using one POC-CCA was significantly higher compared to two Kato-Katz smears (66.1% vs. 28.7%; χ^2^ (1) = 111.7, p < 0.0001).

**Table 2:** Baseline proportion of infected children and intensities of 404 investigated children by six Kato-Katz smears, three POC-CCA tests and gold standard. Gold standard is the combined results of six Kato-Katz smears and three POC-CCAs. The proportion by POC-CCA tests is calculated considering trace as positive (t +). 95% CI = 95% confidence interval, n.a = not applicable.

	Positives		Intensity			
	N	% (95% CI)	Light	Moderate		Heavy
Six Kato-Katz smears	172	42.6 (37.7–47.5)	136 (79.1)	30 (17.4)		6 (3.5)
			Trace	+ 1	+ 2	+ 3
Three POC-CCA (t +)	303	75.0 (70.7–79.3)	132 (43.6)	67 (22.1)	51 (16.8)	53 (17.5)
Gold standard	320	79.2 (75.3–83.1)	n.a.			

Among the 172 children found positive for *S. mansoni* using the Kato-Katz technique, 136 (79.1%), 30 (17.4%) and 6 (3.5%) were classified as having light, moderate and heavy infections, respectively. Of 303 children positive for CCA, 132 (43.6%), 67 (22.1%), 51 (16.8%) and 53 (17.5%) were classified as trace, + 1, + 2 and + 3, respectively. Among 232 individuals tested negative by Kato-Katz, 148 (63.8%) were found positive by CCA. Of those, 103 (69.6%), 25 (16.9%), 17 (11.5%) and 3 (2.0%) were classified as trace, + 1, + 2 and + 3, respectively. On the other hand, among the 101 individuals tested negative by POC-CCA tests, only 17 (16.8%) were diagnosed as positive by Kato-Katz smears with a mean EPG (95% CI) of 39.5 (13.3–65.7).

#### 3.2.2 Diagnostic accuracy of the tests

The agreement between six Kato-Katz smears and the ‘gold standard’ was fair (k = 0.33), and almost perfect for three POC-CCA cassettes (k = 0.88). The sensitivity of three POC-CCAs was higher than that of six Kato-Katz thick smears (94.7%, 95% CI: 92.2–97.2% vs. 53.8%, 95% CI: 48.3–59.3%; p < 0.05).

#### 3.2.3 Day-to-day variability of POC-CCA and Kato-Katz

A total of 308 (76.2%) children scored positive or negative during all three consecutive days using the POC-CCA test; 101 (32.8%) of these were negative for all three days, while 207 (67.2%) were positive for all three days with mean EPG (range) of 54.5 (0–912). However, 96 (23.8%) children had fluctuating results. Changes include any positive reaction (trace, + 1, + 2 or + 3) that changed to negative and *vice-versa*. A total of 52 individuals (54.2%) had negative results for two days and positive for one day with mean EPG (range) of 1.7 (0–56), whereas 44 (45.8%) had negative results for one day and positive results for two days with mean EPG (range) of 8.6 (0–164). The proportion of children that had same scores was significantly higher than the proportion that had different scores (χ^2^ (1) = 110.2; p < 0.001).

Similarly, in 278 (68.8%) children, EPG counts were positive in 46 (16.5%) of the children and negative in 232 (83.5%) of the children for all three consecutive days. However, 126 (31.2%) had fluctuating results as 78 individuals (61.9%) had negative results for two days and positive for one day, whereas 48 (38.1%) had positive results for two days and negative for one day. The proportion of children with homogeneous results was significantly higher than the proportion with heterogeneous results (χ^2^ (1) = 56.4; p < 0.001). Table 3a) and b) shows the day-to-day variability in scores and EPG counts using the POC-CCA and Kato-Katz techniques, respectively. Very few changed from negative/trace/light to + 2/+3/moderate/heavy and *vice-versa*. In Table 4, POC-CCA scores and Kato-Katz intensity categories are compared. A total of 148 (36.6%) individuals scored positive by three POC-CCA test, whereas they were negative according to six Kato-Katz smears. Among those, 103 (69.6%) were scored as ‘trace’. Similarly, 17 (4.2%) individuals were positive by Kato-Katz but negative by POC-CCA. Among those, 15 (88.2%) were of light intensity according to EPG.

**Table 3:** Day-to-day variability in scores using a) POC-CCA test and b) Kato-Katz smears for diagnosis of *S. mansoni* in children from Mwanza Region, Tanzania. Total number of children included = 404 (underlined).

a)												
Day 3							Day 2					
	Neg	Trace	+ 1	+ 2	+ 3	Total	Neg	Trace	+ 1	+ 2	+ 3	Total
*Day 1*												
Neg	111	23	2	1	0	137	113	20	3	0	1	137
Trace	41	56	18	1	0	116	41	59	14	2	0	116
+ 1	2	24	14	16	3	59	6	15	29	8	1	59
+ 2	0	5	17	18	5	45	0	4	18	21	2	45
+ 3	0	0	1	6	40	47	0	0	3	13	31	47
Total	154	108	52	42	48	404	160	98	67	44	35	404

*Day 2*												
Neg	131	27	2	0	0	160						
Trace	22	59	14	3	0	98						
+ 1	1	21	25	17	3	67						
+ 2	0	1	10	19	14	44						
+ 3	0	0	1	3	31	35						
Total	154	108	52	42	48	404						


b)										
Day 3						Day 2				
	Neg	Light	Moderate	Heavy	Total	Neg	Light	Moderate	Heavy	Total
*Day 1*										
Neg	249	29	7	3	288	255	25	5	3	288
Light	44	36	4	0	84	52	28	3	1	84
Moderate	5	7	13	1	26	7	7	12	0	26
Heavy	0	1	3	2	6	0	2	0	4	6
Total	298	73	27	6	404	314	62	20	8	404

*Day 2*										
Neg	270	37	6	1	314					
Light	26	31	5	0	62					
Moderate	1	4	14	1	20					
Heavy	1	1	2	4	8					
Total	298	73	27	6	404

**Table 4:** Comparison of the results for POC-CCA test scores (in band intensity categories; negative, trace, + 1, + 2 and + 3) and Kato-Katz smears (in egg intensity categories; negative, light, moderate and heavy) for samples from three consecutive days (total numbers in bold).

	POC-CCA intensity categories					
Kato-Katz intensity categories	negative	Trace	+ 1	+ 2	+ 3	Total
Negative	84	103	25	17	3	**232**
Light (1–99 EPG)	15	26	39	26	30	**136**
Moderate (100–399 EPG)	2	2	3	8	15	**30**
Heavy (≥ 400 EPG)	0	1	0	0	5	**6**
Total	**101**	**132**	**67**	**51**	**53**	**404**

#### 3.2.4 Correlation between pixel density and CCA scores or EPG

**Fig. 3:**
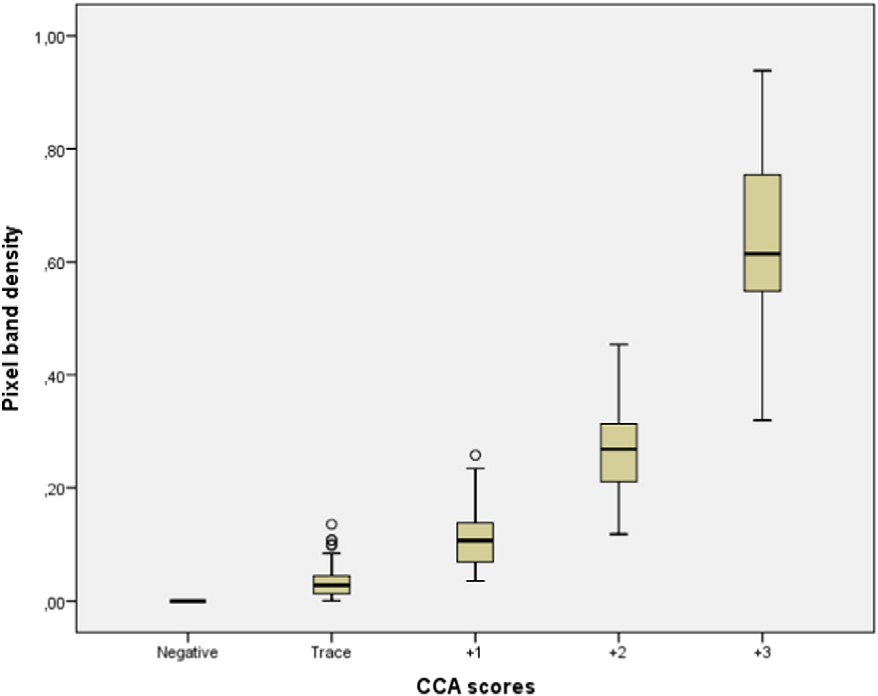
Correlation between pixel band intensity for three days (medians) and visual CCA scores (mean for three POC-CCA tests; scores: negative, trace, + 1, + 2 and + 3).

**Fig. 4:**
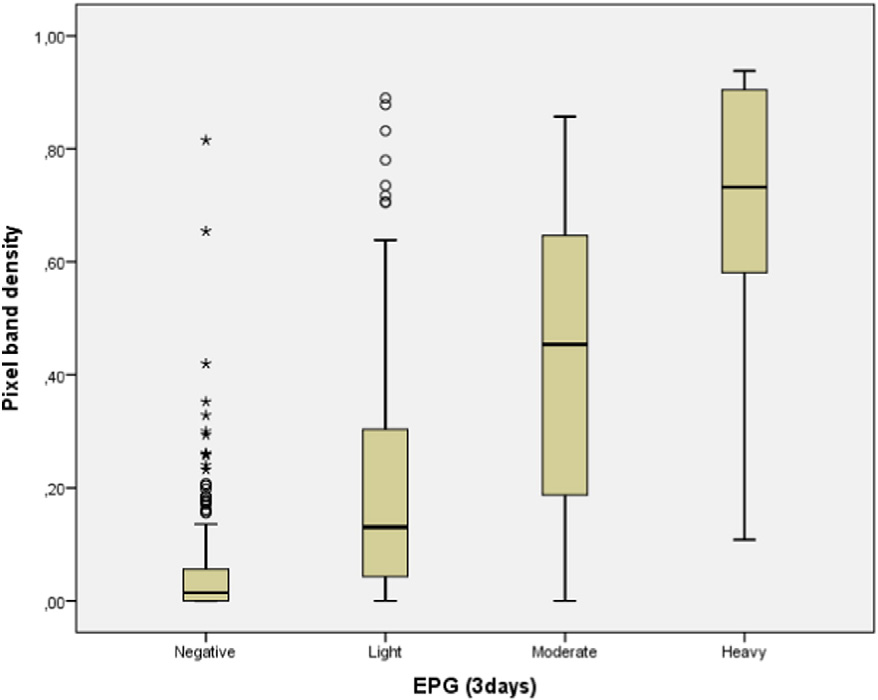
Correlation between pixel band intensity for three days (medians) and EPG (mean for six Kato-Katz smears in egg intensity categories: negative (0 EPG), light (1–99 EPG), moderate (100–399 EPG) and heavy (≥ 400 EPG) infections).

A total of 404 children provided a complete set of three POC-CCA tests. A total of 761 cassettes were positive and therefore visually read and photographed. The strength of the association between pixel band density and POC-CCA scores shows a positive and very strong correlation (pixel median = 0.03, 0.11, 0.27 and 0.61 for CCA score = trace, + 1, + 2 and + 3, respectively; r = 0.91; p < 0.0005) between variables, the stronger the colour of the test band the higher CCA scores and *vice-versa* (Fig. 3). Also, the strength of the association between pixel band density and egg counts (EPG) in intensity categories shows a positive and moderate correlation (pixel median = 0.13, 0.45 and 0.73 for EPG categories = light, moderate and heavy, respectively; r = 0.56; p-value < 0.0005), the higher pixel density the higher egg counts and *vice-versa* (Fig. 4).

## 4 Discussion

The number of *S. mansoni* infected children determined by POC-CCA assay of one urine on three consecutive days was significantly higher (p < 0.0005) than the number determined by duplicate Kato-Katz thick smears of one stool sample on the same three days, which is in agreement with previous studies (Erko et al., 2013, Coulibaly et al., 2013, Colley et al., 2013, Mwinzi et al., 2015). It is also in accordance with a recent systematic review which found that below 50% prevalence by Kato-Katz, the prevalence by the POC-CCA assay was between 1.5- and 6-fold higher than prevalence by Kato-Katz, while the two methods yielded approximately the same prevalence when prevalence was above 50% by Kato-Katz (Kittur et al., 2016).

The Kato-Katz technique has been the diagnostic method for detection of intestinal schistosomiasis in epidemiological studies for decades (Katz et al., 1972, Coulibaly et al., 2013). However, it has low sensitivity even when multiple samples are tested (Shane et al., 2011) making it less useful in areas with low intensity infections (Coulibaly et al., 2013). The low sensitivity is due to the relatively small stool sample investigated, the daily fluctuations in egg excretion and the heterogeneous distribution of eggs within the stool sample (Berhe et al., 2004, Kongs et al., 2001). Furthermore, there is no excretion of eggs from immature worms and in single sex infections and these are therefore missed by the Kato-Katz technique whereas all schistosome worms excrete CCA. These factors in conjunction may explain the much higher sensitivity of the POC-CCA assay compared to the Kato-Katz technique. The combination of results by six Kato-Katz thick smears and triple POC-CCA tests as ‘gold standard’ in order to assess the sensitivity of the tests might not be the most optimal ‘gold standard’ to use. Faecal PCR has been shown to be a very sensitive method (Abdel-Hafeez et al., 2015) and could be a good alternative for further sensitivity investigations.

Cost is an important issue in schistosomiasis endemic countries. In a recent study in Kenya, the cost of a single POC-CCA was compared to two and six Kato-Katz smears, respectively. Comparing costs divided in labour, supplies, capital, transportation and overhead showed that using a single POC-CCA was only slightly more expensive than using two Kato-Katz smears (Worrell et al., 2015). This might be justified with the higher sensitivity of the POC-CCA and by the fact that the cost of the CCA tests probably decreases in the future. A drawback though is that the POC-CCA test cannot detect soil transmitted helminthiasis as the Kato-Katz method can.

There were 148 (63.8%) of the egg negative children who were CCA positives, which can be explained by the reasons mentioned above. On the other hand, it is more difficult to explain why 17 (9.9%) egg positive individuals showed negative results with the POC-CCA test (Erko et al., 2013). We cannot completely rule out that mistake happens when collecting and processing hundreds of samples in the field resulting in a mismatch between a stool sample and its supposedly corresponding urine sample. Although unlikely, this can be due to sharing of stool and/or urine samples between study participants, or mislabelling during sample collection and processing. Nevertheless, negative CCA results in those excreting eggs detected by Kato-Katz, might raise concerns about the accuracy of the POC-CCA for diagnosing *S. mansoni* infections and needs to be investigated further.

The software tool (Image Studio Lite®) was able to read all bands from positive POC-CCA assays (including the ‘trace’ values) and could thus be an important tool for optimizing the use of the POC-CCA test by overcoming reading-related difficulties and inter-observer variations. It is promising that there was a positive and strong correlation between pixel quantification and visual scores of the result bands of the POC-CCA tests and a positive but less strong correlation between pixel quantification of the CCA result bands and the mean of EPG determined by six Kato-Katz smears. This is in accordance with other previous findings between EPG and intensity of the POC-CCA (Shane et al., 2011).

A contentious issue in the use of the POC-CCA has been the interpretation of the result band and its inter-reader variability, especially when the result is a ‘trace’ (Standley et al., 2010, Stothard et al., 2006, Stothard et al., 2011). However, the reading of the test band by the computer is time consuming and the results depend on the quality of the picture and the reader's ability with the computer. It is also demanding to standardize the conditions for taken of pictures. In addition, without any exact determination of the POC-CCA intensity scale, it is difficult to interpret results. Better training in reading POC-CCA or an addition of a comparison line on the assay has been suggested to overcome this (Colley et al., 2013).

Computer and camera are necessary for quantification of the POC-CCA assay and might not be feasible in some settings where *S. mansoni* is endemic and therefore less practical as a POC tool. As has already been suggested by Mudanyali et al. (2012), the use of a RDT (Rapid Diagnostic Test) reader application run by a cell phone has shown its efficiency and this could overcome the computer–camera obstacle by reading the band automatically. However, further investigation should clarify whether a phone application could be developed to read and quantify the CCA bands.

There was some day-to-day variability for both techniques; 23.8% (96) of the tested individuals showed non-uniform results with respect to CCA scores, compared to 31.2% (126) based on egg counts by the Kato-Katz method. A possible explanation for the variability of POC-CCA assay results could be attributed to some day-to-day fluctuation of CCA levels in urine as previously studied suggesting the necessity for collecting several samples on different days for more reliable diagnosis of *S. mansoni* (van Etten et al., 1996, Degarege et al., 2014). In agreement with the present study, other studies have reported daily fluctuation in CCA observed mainly in children with very light infection (Erko et al., 2013, Adriko et al., 2014, Tchuem Tchuenté et al., 2012).

In the small pilot study, 90 out of 106 participants (84.9%) did not change POC-CCA scores from *morning* to *midday* urine, but 13 (12.3%) individuals changed from negative to positive and 3 (2.8%) from positive to negative. It has previously been reported that CCA variability could be attributed to the day-to-day fluctuation of antigen levels in urine (Polman et al., 1998) and that results from the antigen determinations in urine should be interpreted with caution, since concentrations might vary considerably depending on fluid intake, kidney-associated and general morbidity (Shane et al., 2011, van Dam et al., 1996). We asked the children for their first urine in the morning, but we could not control whether they took some fluid before. Neither was food, exercise nor morbidity controlled. In spite of this, our results indicate that time of day for collection of urine has no major importance.

In conclusion, the current study showed that even a single POC-CCA test seems to be a more appropriate tool for *S. mansoni* diagnosis compared to six Kato-Katz smears in endemic communities such as Mwanza Region. Although the diagnostic tool also has its limitations such an inter-reader variability of the CCA bands, optimization of the test could be assisted by computer software (Image Studio Lite®). This software quantifies the test colour band and indicates a measure of infection intensity in a quantifiable and objective way, thus avoiding reader-variability. Importantly, the software is able to read the ‘trace’ values, but more investigations are needed to determine how well the tool can distinguish between negative and ‘trace’ values and whether development of an application for smartphones is possible and beneficial. In terms of urine collection, collection time seems not to be an influential factor for detection of the antigen.

## Competing interests

The authors declare that they have no competing interests.
